# Nitazoxanide synergizes polymyxin B against *Escherichia coli* by depleting cellular energy

**DOI:** 10.1128/spectrum.00191-24

**Published:** 2024-06-21

**Authors:** Xuejia Jiang, Dongliang Chen, Xiaoyang Wang, Chunmei Wang, Haihong Zheng, Wenchong Ye, Wen Zhou, Guoping Liu, Keyu Zhang

**Affiliations:** 1 Key Laboratory of Veterinary Chemical Drugs and Pharmaceutics, Ministry of Agriculture and Rural Affairs, Shanghai Veterinary Research Institute, Chinese Academy of Agricultural Sciences, Shanghai, China; 2 College of Animal Science, Yangtze University, Jingzhou, Hubei, China; University of Torino, Turin, Italy

**Keywords:** antibiotic adjuvant, nitazoxanide, antibiotic resistance, gram-negative bacteria, polymyxin B

## Abstract

The rapid expansion of antibiotic-resistant bacterial diseases is a global burden on public health. It makes sense to repurpose and reposition already-approved medications for use as supplementary agents in synergistic combinations with existing antibiotics. Here, we demonstrate that the anthelmintic drug nitazoxanide (NTZ) synergistically enhances the effectiveness of the lipopeptide antibiotic polymyxin B in inhibiting gram-negative bacteria, including those resistant to polymyxin B. Mechanistic investigations revealed that nitazoxanide inhibited calcium influx and cell membrane depolarization, enhanced the affinity between polymyxin B and the extracellular membrane, and promoted intracellular ATP depletion and an increase in reactive oxygen species (ROS), thus enhancing the penetration and disruption of the *Escherichia coli* cell membrane by polymyxin B. The transcriptomic analysis revealed that the combination resulted in energy depletion by inhibiting both aerobic and anaerobic respiration patterns in bacterial cells. The increased bactericidal effect of polymyxin B on the *E. coli ∆nuoC* strain further indicates that NuoC could be a promising target for nitazoxanide. Furthermore, the combination of nitazoxanide and polymyxin B showed promising therapeutic effects in a mouse infection model infected with *E. coli*. Taken together, these results demonstrate the potential of nitazoxanide as a novel adjuvant to polymyxin B, to overcome antibiotic resistance and improve therapeutic outcomes in refractory infections.

**IMPORTANCE:**

The rapid spread of antibiotic-resistant bacteria poses a serious threat to public health. The search for potential compounds that can increase the antibacterial activity of existing antibiotics is a promising strategy for addressing this issue. Here, the synergistic activity of the FDA-approved agent nitazoxanide (NTZ) combined with polymyxin B was investigated *in vitro* using checkerboard assays and time-kill curves. The synergistic mechanisms of the combination of nitazoxanide and polymyxin B were explored by fluorescent dye, transmission electron microscopy (TEM), and transcriptomic analysis. The synergistic efficacy was evaluated *in vivo* by the *Escherichia coli* and mouse sepsis models. These results suggested that nitazoxanide, as a promising antibiotic adjuvant, can effectively enhance polymyxin B activity, providing a potential strategy for treating multidrug-resistant bacteria.

## INTRODUCTION

Antibiotics are indispensable pharmaceuticals for the treatment of severe bacterial infections in humans and animal husbandry. Unfortunately, the emergence and rapid dissemination of bacterial pathogens that are resistant to current antibiotics in hospitals, community settings, and animal husbandry pose an increasing threat to global health (1–3). It is concerning that the incidence of multidrug resistance (MDR), extensive-drug-resistant (XDR), or pan-drug resistance (PDR) among clinical isolates is increasing every year (4, 5). Gram-negative bacteria are inherently resistant to many antibiotics because of a dual-membrane envelope that prevents many antibiotics from reaching their targets, resulting in infections caused by gram-negative bacteria that are arguably more difficult to treat (6). The development process of novel antimicrobial drug entities has significantly lagged behind the evolution speed of drug-resistant bacteria. Therefore, there is an urgent need to explore other new strategies to combat infections (7, 8).

Polymyxins, such as polymyxin B and colistin, are cationic lipopeptides that exhibit a strong positive charge. They primarily exert their antimicrobial activity against gram-negative bacteria by tightly binding to the lipid A component of lipopolysaccharides within the outer membrane of the cellular envelope, ultimately leading to bacterial cell death (9). The emergence of carbapenem-resistant Enterobacteriaceae strains has significantly compromised the efficacy of available antimicrobial agents, thereby highlighting the potential therapeutic value of polymyxin antibiotics. Over recent years, polymyxins have gained widespread recognition as “last-resort” antibiotics for treating infections caused by multidrug-resistant and extensively drug-resistant gram-negative bacteria, particularly Enterobacterales, *Acinetobacter baumannii*, and *Pseudomonas aeruginosa* (10, 11). However, the rapid dissemination of *mcr-1* gene poses an additional threat to the effectiveness of polymyxin antibiotics (12). To address this escalating resistance issue among gram-negative bacteria toward conventional antibiotics, we are exploring antibiotic adjuvant strategies aimed at enhancing antibiotic efficacy and prolonging the lifespan of existing antibacterial agents. Previous studies have demonstrated that niclosamide (13), robenidine (14), miconazole (15), SLAP-S25 (16), compound 666-15 (17), along with certain antibiotics like rifampicin, can synergistically enhance the effectiveness of polymyxin B against gram-negative bacteria (18).

Nitazoxanide (NTZ) was developed as an oral antiparasitic agent through scaffold modification of the anthelmintic niclosamide (19). The U.S. Food and Drug Administration (FDA) has approved nitazoxanide for the treatment of diarrhea caused by *Cryptosporidium parvum* and *Giardia intestinalis*. Nitazoxanide has also been licensed in Bangladesh, Egypt, India, and Latin America, where it is widely commercialized as an oral broad-spectrum antiparasitic agent for treating intestinal protozoa and helminths (20). Over the years, numerous studies have demonstrated that nitazoxanide exhibits antiviral properties (20, 21), possesses potential anti-tumor effects (22), and displays anti-inflammatory activity (23). It induces autophagy (24) and may have applications in diabetes treatment (25) as well as in Alzheimer’s disease therapy (26, 27). Additionally, nitazoxanide has demonstrated activity against a wide range of obligate and facultative anaerobic gram-positive and gram-negative bacteria *in vitro*. However, its effectiveness against members of the Enterobacteriaceae family and Pseudomonas species under aerobic conditions is limited (28). Furthermore, it remains unclear whether nitazoxanide can serve as an adjuvant to antibiotics to enhance their bactericidal activity.

To investigate the efficacy of antibiotic combinations, we evaluated the synergistic activity of several commercially approved antibiotics in combination with nitazoxanide against *Escherichia coli* (*E. coli*). Our findings demonstrated that nitazoxanide significantly potentiates the antimicrobial activity of polymyxin B against multiple gram-negative pathogens. Subsequent investigations revealed that nitazoxanide enhances bacterial membrane permeability and disrupts the electrochemical potential in *E. coli* cells. Notably, nitazoxanide also interferes with cellular oxidative phosphorylation, leading to a substantial reduction in ATP levels and thereby augmenting the bactericidal effect of polymyxin B. Collectively, these results underscore the potential utility of nitazoxanide as an adjunctive therapy to polymyxin B for treating bacterial infections.

## RESULTS

### Nitazoxanide potentiates polymyxin B against gram-negative bacteria

Nitazoxanide did not exhibit any antibacterial effects against gram-negative bacterial strains such as *E. coli*, *Salmonella enterica* serovar Typhi *(S.* Typhi), *Proteus mirabilis* (*P. mirabilis*), and *Klebsiella pneumoniae* (*K. pneumoniae*) at the highest concentrations (128 µg/mL) tested. To investigate potential synergistic combinations against these strains, checkerboard dilution assays were conducted with nitazoxanide and various classes of antibiotics, including β-lactams, polymyxins, aminoglycosides, tetracyclines, fluoroquinolones, macrolides, sulfonamides, lincomycins, and rifamycins. The screening revealed that only polymyxin B exhibited synergistic activity with nitazoxanide against *E. coli*, while other commonly used antibiotics did not demonstrate synergism with nitazoxanide.

**Fig 1:**
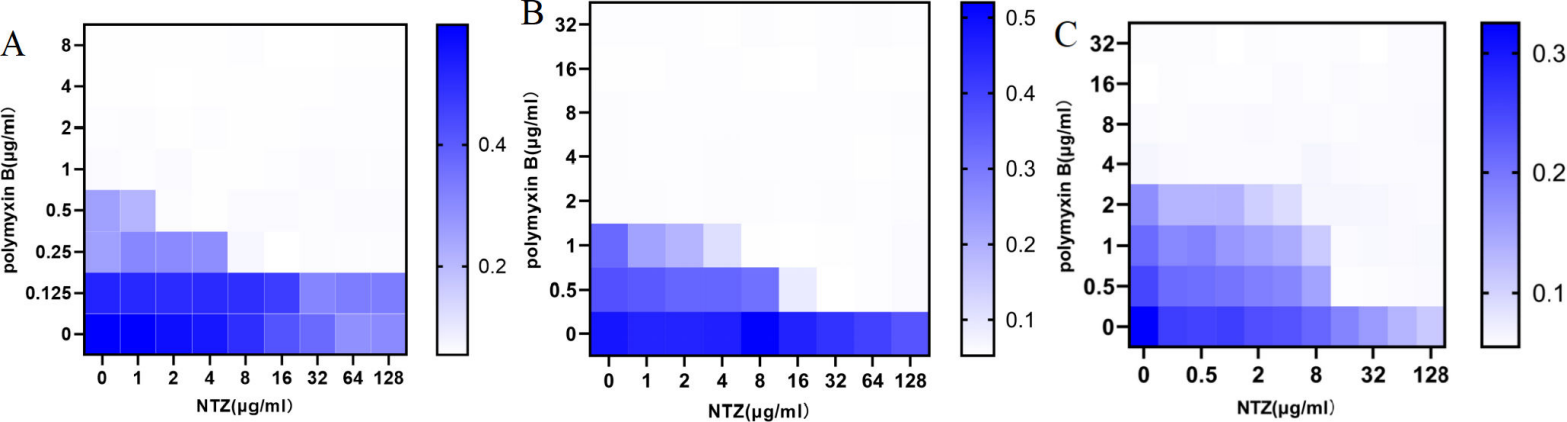
NTZ drastically potentiates polymyxin B activity against *E. coli*. Checkerboard broth microdilution assays between nitazoxanide and polymyxin B against *E. coli* ATCC 25922 (**A**), colistin-resistant strain B2 (**B**), and *S.* Typhi Sa6 (**C**). Dark‐blue regions represent higher cell density and lower inhibition rate of combinational treatment. Data represent the mean OD (600 nm) of two biological replicates. *x*‐ and *y*‐axes of figures are presented as log2 scale. Synergy is defined as an FIC index of ≤0.5.

Although most isolates of *E. coli*, *S.* Typhi, *P. mirabilis*, and *K. pneumoniae* from animals were resistant to polymyxin B, the combination of polymyxin B and nitazoxanide showed a synergistic effect against the standard strain of *E. coli* ATCC 25922 as well as against isolated strains (Fig. 1A through C). The results of minimum inhibitory concentration (MIC), fractional inhibitory concentration (FIC), and dose reduction index (DRI) values for the combination of polymyxin B and nitazoxanide against gram-negative bacteria are presented in Table 1. The DRI values of polymyxin B were significantly increased in the presence of nitazoxanide, ranging from 4- to 16-fold.

**TABLE 1:** The MIC values for nitazoxanide and polymyxin B, as well as the combined effect of nitazoxanide on the MIC of polymyxin B against gram-negative strains

Organism	Strains	MIC (μg/mL)			Combination effect	
		Single drug		Combination	FIC^*c*^	DRI^*d*^
		NTZ^*a*^	PMB^*b*^	NTZ:PMB		NTZ:PMB
*E. coli*	ATCC 25922	>128	1	8:0.25	Synergism (0.3125)	16:4
	B2	>128	2	16:0.5	Synergism (0.375)	8:4
	5E	>128	4	16:0.25	Synergism (0.1875)	8:16
	7E	>128	4	16:0.5	Synergism (0.25)	8:8
	8E	>128	4	16:0.5	Synergism (0.25)	8:8
*S.* Typhi	S3	>128	32	8:8	Synergism (0.3125)	16:8
	S6	>128	4	16: 0.5	Synergism (0.25)	8:8
	S9	>128	2	16: 0.5	Synergism (0.375)	8:4
	S21	>128	32	8:8	Synergism (0.3125)	16:4
*K. pneumoniae*	K3	>128	4	32:1	Synergism (0.5)	4:4
*P. mirabilis*	P3	>128	16	32:4	Synergism (0.5)	4:4

^*a*^ NTZ, nitazoxanide.

^*b*^ PMB, polymyxin B.

^*c*^ The results indicate synergism when the corresponding FIC ≤ 0.5; additivity when 0.5 < FIC ≤ 1, indifference when 1 < FIC ≤ 4 and antagonism when the FIC > 4.

^*d*^ DRI, dose reduction index.

**Fig 2:**
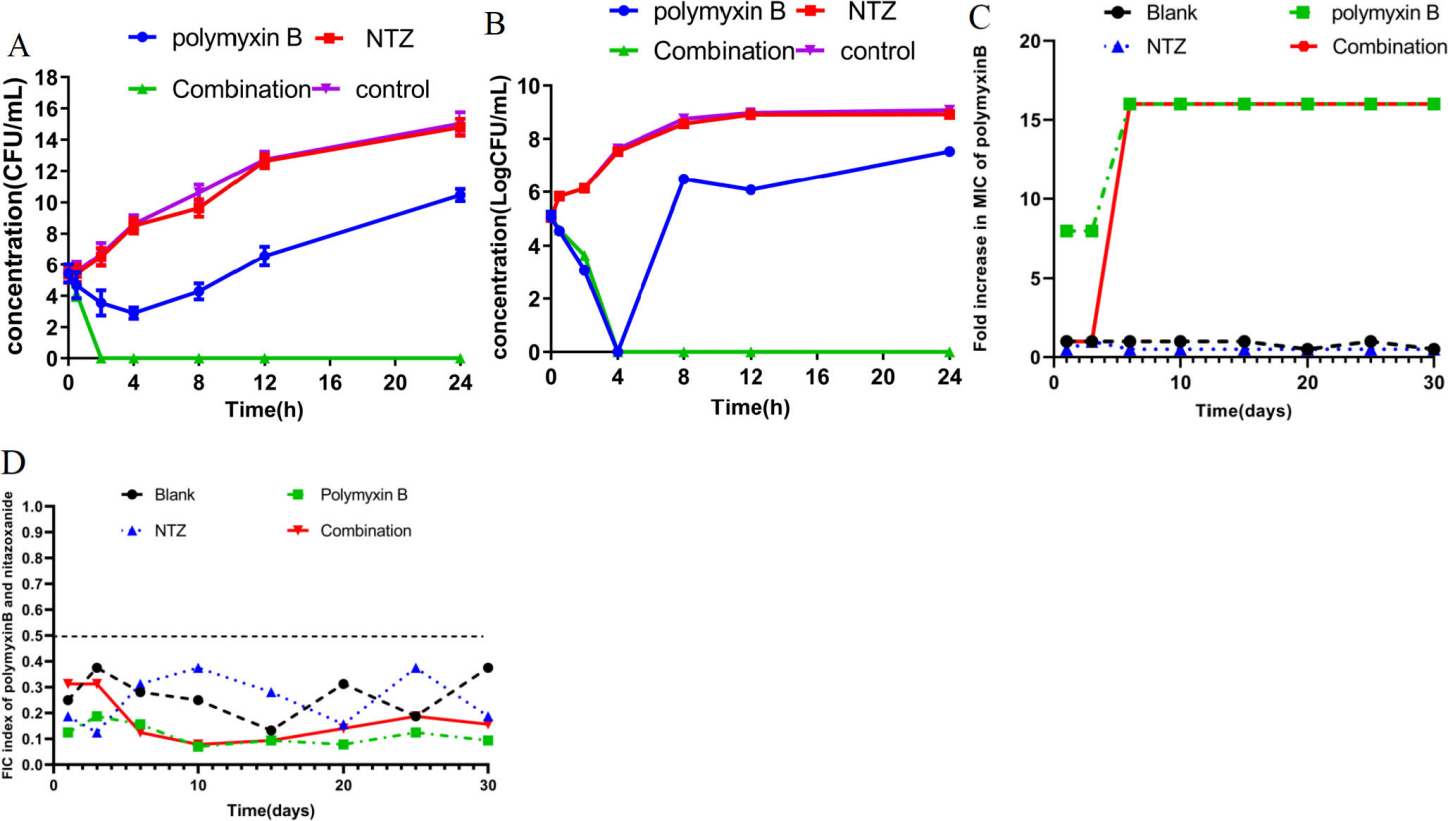
Time‐dependent killing of pathogens by the combination of polymyxin B and NTZ. (**A**) *E. coli* ATCC 25922 was grown in MHB broth then treated with PBS, polymyxin B (polymyxin, 0.125 µg/mL), or NTZ (8 µg/mL) alone or in combination (0.125 µg/mL polymyxin + 8 µg/mL NTZ). (**B**) *E. coli* B2 were grown in MHB broth then treated with PBS, polymyxin B (polymyxin B, 2 µg/mL), or NTZ (16 µg/mL) alone or in combination (2 µg/mL polymyxin + 16 µg/mL NTZ). The bacterial CFUs per milliliter at different time points during 24 h were determined. All experiments were performed three times, and the mean ± SD is shown. (**C**) The addition of nitazoxanide retards the evolution of polymyxin B resistance to *E. coli* ATCC 25922 *in vitro*. (**D**) Nitazoxanide and polymyxin B still have a synergistic effect (FIC ≤ 0.5) on the drug-resistant strains induced through passage.

The analysis of the checkerboard assay indicated that nitazoxanide enhanced the antibacterial activity of polymyxin B, and this enhancement was further validated by time-killing curve testing at sub-MIC levels for *E. coli* ATCC 25922 and B2 (Fig. 2A and B). In the control group, the bacterial growth of both standard and isolated strains was observed to be nearly equivalent to that of the sample treated with nitazoxanide alone. Treatment with polymyxin B alone at sub-MIC levels resulted in a reduction in colony count of ATCC 25922 and B2 within 24 h compared to the control growth and nitazoxanide alone. A consistent reduction of at least five log10 was still evident after 4 h of incubation, although the number of bacteria present had increased thereafter. This reduction remained consistent compared to the growth control and nitazoxanide alone. It is noteworthy that the combination of polymyxin B and nitazoxanide significantly reduced the colony count of the standard and isolated strains over 2 and 4 h, demonstrating a synergistic effect compared to the control growth and polymyxin B alone. After 2 and 4 h, bacteria were eliminated in both ATCC 25922 and B2, respectively.

To get a better understanding of nitazoxanide on the development of polymyxin B resistance, we performed serial passages of *E. coli* ATCC 25922 with polymyxin B in the presence and absence of nitazoxanide during 30 d. MIC increase and FIC index for calculated passages are presented in Fig. 2C and D. Both groups treated with polymyxin B alone or in combination produced high-resistant strains to polymyxin B. However, the combination of polymyxin B and nitazoxanide still had a synergistic effect (FIC ≤ 0.5) on all passaged *E. coli* including these resistant strains.

### Nitazoxanide promotes polymyxin B to disrupt the integrity of *E. coli* membranes

**Fig 3:**
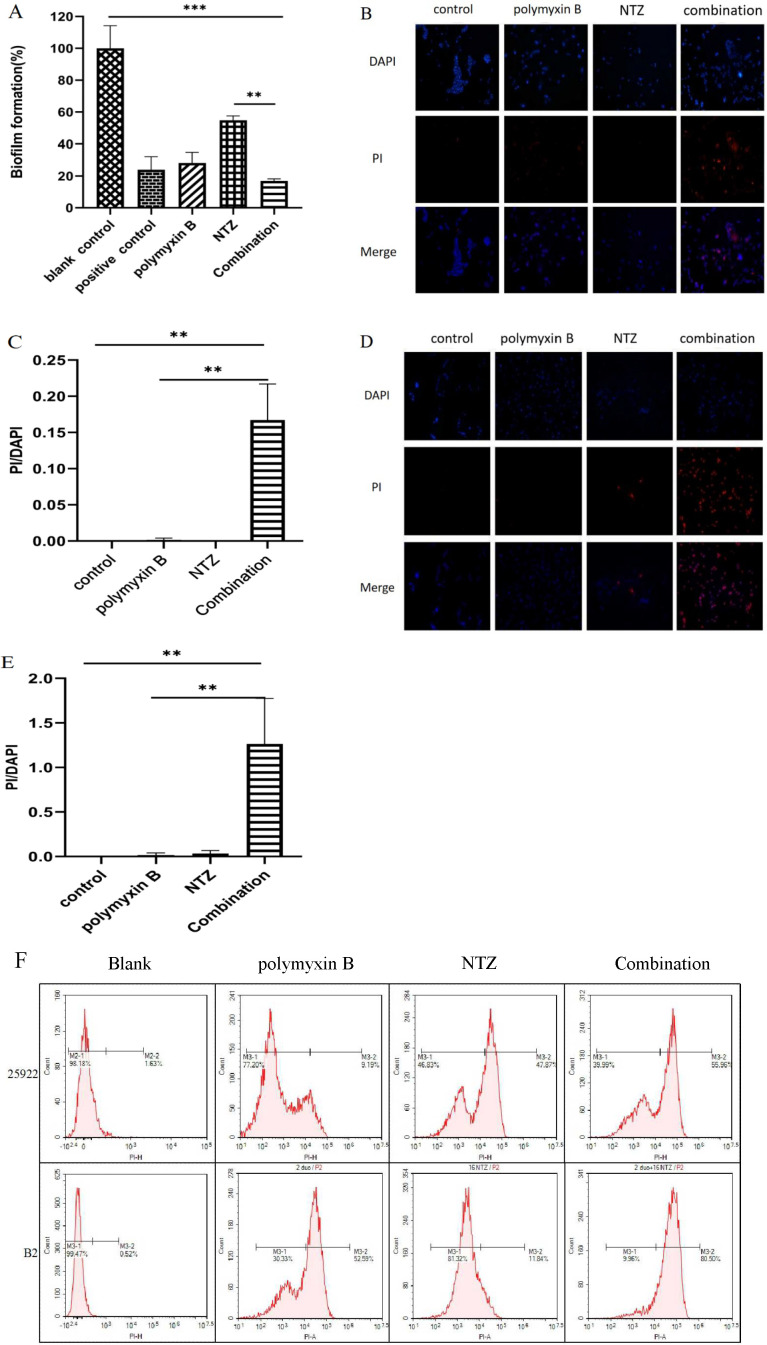
NTZ promotes polymyxin B to disrupt the integrity of *E. coli* membranes. (**A**) NTZ enhances the inhibitory effect of polymyxin B on biofilm formation. The concentrations of polymyxin B group, NTZ group, and combination group were 0.5, 32, and 0.5 µg/mL + 32 µg/mL, biofilm formation = OD_drug_/OD_blank_ × 100%. Nitazoxanide enhanced the damage of polymyxin B on the outer membrane of *E. coli* ATCC25922 (**B**) and B2 (**D**), and promoted the fluorescence intensity of intracellular PI. (**C**) and (**E**) are histograms for quantitative fluorescence intensity analysis of PI in *E. coli* ATCC25922 and B2 cells, respectively. The concentrations of polymyxin B group, NTZ group, and combination group for *E. coli* ATCC25922 strain were 1, 128, and 1 µg/mL + 128 µg/mL, and for *E. coli* B strain were 8, 32, and 8 µg/mL + 32 µg/mL, respectively. (**F**) Flow cytometry assay demonstrated that NTZ enhanced the proportion of intracellular PI-stained cells in *E. coli* treated with polymyxin B. The concentrations of polymyxin B group, NTZ group, and combination group for *E. coli* ATCC25922 strain were 0.125, 16, and 0.125 µg/mL + 16 µg/mL, and for *E. coli* B strain were 2, 16, and 2 µg/mL + 16 µg/mL, respectively.

The activities of polymyxin B in the presence or absence of nitazoxanide against biofilm formation of *E. coli* ATCC 25922 were texted by the optical density (OD) ratio of bacterial biofilms stained with crystal violet between drug treatment and blank control. As shown in Fig. 3A, all drug treatment groups showed significant inhibition of bacterial biofilm formation compared to the blank control. The biofilm formation activity of polymyxin B at a concentration of 0.5 µg/mL was 28.2%, exhibiting a strong inhibitory activity of biofilm formation compared to the blank control as well as comparable to the positive control of 70% propan-2-ol solution (23.9%). Additionally, nitazoxanide reduced almost 50% of the biofilm at a concentration of 32 µg/mL. Interestingly, the combination of polymyxin B and nitazoxanide (0.5 µg/mL + 32 µg/mL) significantly further reduced biofilm formation to 16.9%, demonstrating the potential for synergistic inhibition of biofilm formation.

Propidium iodide (PI) is a non-permeable fluorochrome that accumulates only in cells with damaged membranes, providing a measure of membrane integrity. The fluorescence signals of PI uptake by *E. coli* observed under fluorescence microscopy are shown in Fig. 3B through E. The uptake of PI was weak in both *E. coli* ATCC 25922 and B2 treated with polymyxin B at concentrations of 1 and 8 µg/mL, respectively. Nitazoxanide-alone treatment also showed less significant uptake of PI. Interestingly, the combination of polymyxin B and nitazoxanide exhibited a more robust in fluorescent signaling of PI than polymyxin B or nitazoxanide alone in both *E. coli* ATCC 25922 and B2. The results of flow cytometry assay for PI uptake by *E. coli* are shown in Fig. 3F. The fluorescence abundance of PI by polymyxin B-treated *E. coli* ATCC 25922 and B2 was 9.19% and 52.59%, respectively. This abundance was 47.87% and 11.84% in the nitazoxanide-treated group; 55.96% and 80.50% in the combined treatment group, respectively. The synergy results showed that the fluorescence abundance was higher when the two drugs were used together than when they were used alone, which further illustrated the results of bacterial permeability. The results of the flow cytometry assay for bacterial permeability were consistent with the fluorescence microscopy observation that the combination of polymyxin B and nitazoxanide disrupted cell membrane integrity more than the treatment with polymyxin B or nitazoxanide alone.

The swimming motility of *E. coli* was assessed by measuring the colony diameter after exposure to the tested substances or their combination for 24, 36, and 48 h. In soft agar chemotaxis assays, polymyxin B at a concentration of 1/2 MIC, which effectively inhibited biofilm formation, did not impact swarming motility. Nitazoxanide treatment did not significantly reduce colony diameter either. Furthermore, there was no observed effect on bacterial motility when polymyxin B and nitazoxanide were combined (data not shown).

**Fig 4:**
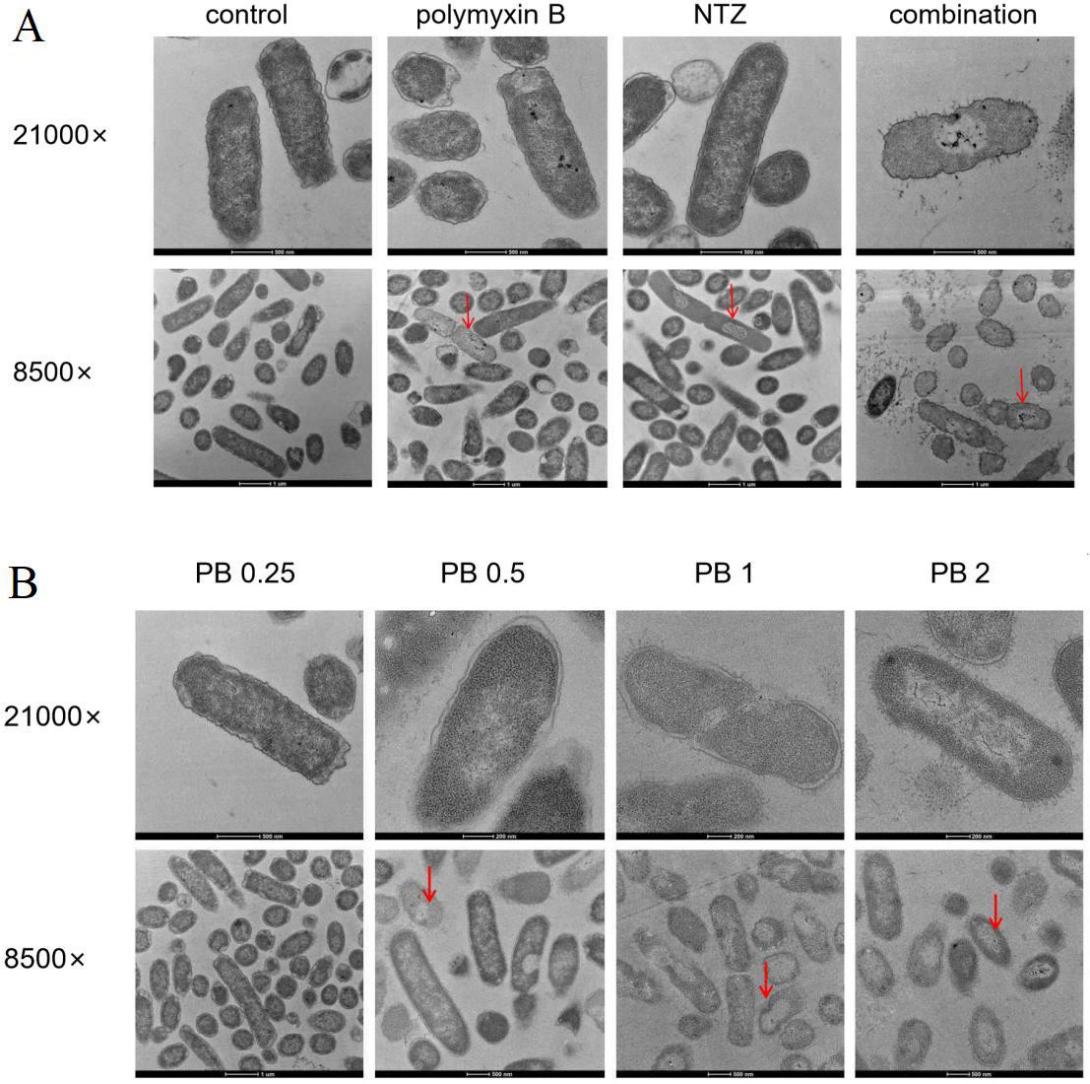
Morphology of *E. coli* ATCC 25922 was investigated by TEM after treatment of polymyxin B, NTZ alone, or the combination for 2 h, which revealed that the combination had greater damage to *E. coli* cells than that of high-dose polymyxin B. (**A**) CON, control group; PB, 0.5 µg/mL polymyxin; NTZ, 64 µg/mL nitazoxanide; COM, 0.5 µg/mL polymyxin B + 64 µg/mL NTZ. Note short filamentous appendages radiating from the outer membrane, thicker envelope, electron transparent, but clumped cytoplasm, mesosome. (**B**) PB 0.25, PB 0.5, PB 1, and PB 2 were treated with 0.25, 0.5, 1.0, and 2.0 µg/mL polymyxin B, respectively. The arrow points to the obvious damage site. TEM results revealed that the combination of NTZ and polymyxin B had greater damage to *E. coli* cells than that of high-dose polymyxin B.

Morphological changes associated with treatment in *E. coli* were assessed by visual comparison using transmission electron microscopy (TEM). As shown in Fig. 4A and B, the cell envelope of *E. coli* in untreated controls was clearly visible as plasma membrane, cell wall, and capsule. The regions with high and low electron density could be distinguished in the cytoplasmic substructure. Compared with the control group, the regions with lower electron density in cytoplasmic substructures were more pronounced in nitazoxanide-treated cells, but the cell membrane morphology was integrated and smooth. In contrast, *E. coli* cells exposed to polymyxin B presented a swollen envelope with tubular-like radiant appendages. The plasma membrane was partly detached from the cell wall. The electron density in cytoplasm and envelope was reduced and almost electron transparent. The morphological changes of membrane disruption and cytoplasmic leakage in *E. coli* became more severe with increasing doses of polymyxin B. Polymyxin B group (2 µg/mL) exhibited bacterial fragments and perforations; membrane debris was found surrounding the cells (Fig. 4B). Of note, the cells treated with the combination of polymyxin B and nitazoxanide exhibited distinct coronate tubular appendages. The gap between the cell wall and the cytoplasmic membrane disappeared, and electron dense material in cytoplasm was reduced and almost electron transparent. A large amount of membrane debris was found around the cells. The damage to the *E. coli* cells was more powerful with their combination than with high doses of polymyxin B.

**Fig 5:**
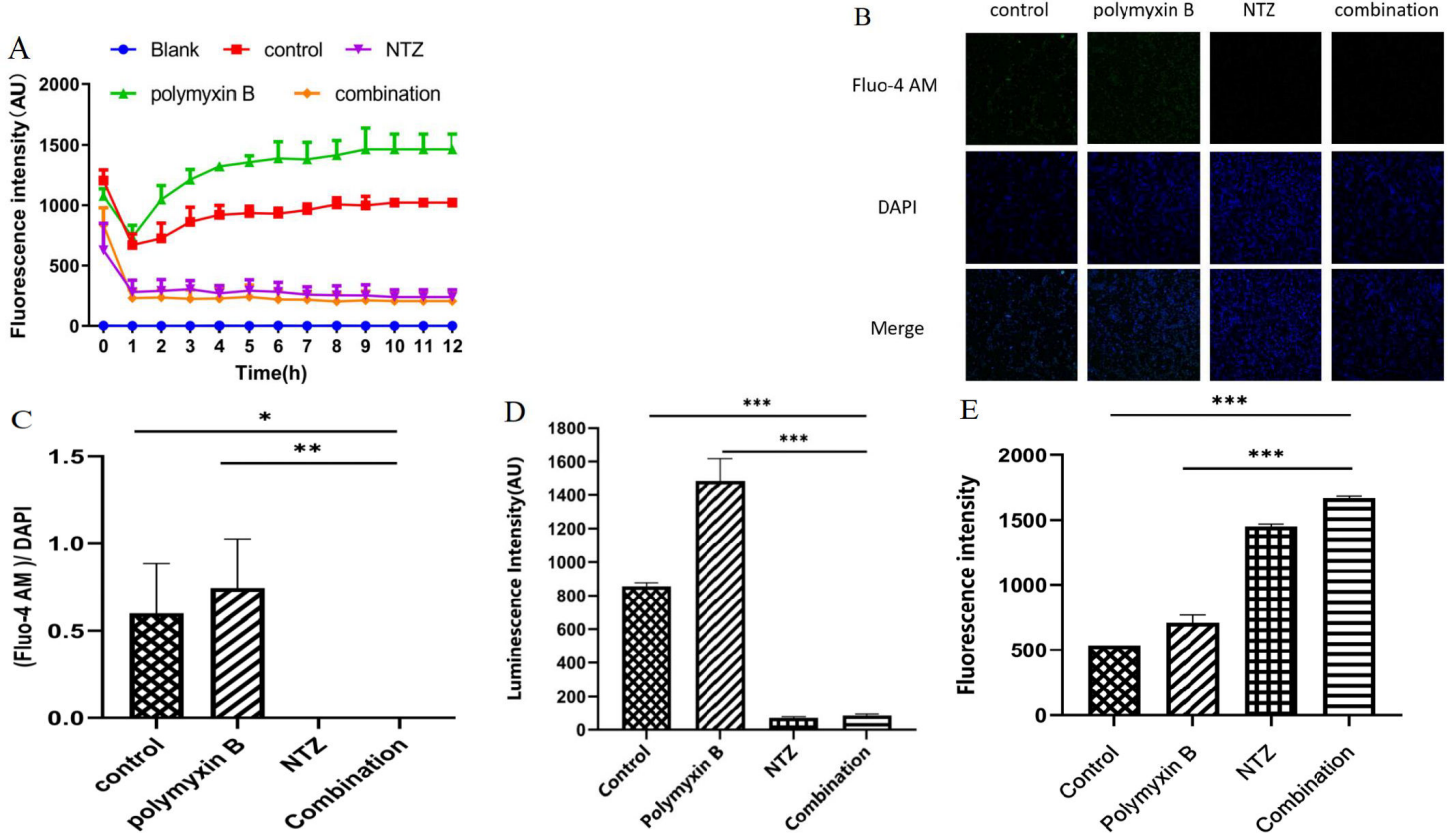
Synergistic mechanisms of polymyxin B–NTZ combination. (**A**) NTZ and the combination enhanced the membrane potential of *E. coli* ATCC 25922, as monitored by DiSC3(5). (**B**) The fluorescence intensity of the Fluo-4 AM probe observed under fluorescence microscope, and (**C**) the fluorescence intensity ratio histogram of Fluo-4 AM/DAPI; Fluo-4 AM is intracellular calcium ion concentration, DAPI is a popular chromosome counterstain. These results showed that NTZ and the combination inhibited intracellular calcium ion concentration. (**D**) Decreased production of intracellular ATP in *E. coli* cells treated with NTZ or the combination. (**E**) ROS production was significantly increased in *E. coli* cells treated with polymyxin B, NTZ, and the combination. The concentrations of polymyxin B group, NTZ group, and combination group for *E. coli* ATCC25922 strain were 1, 16, and 1 µg/mL + 16 µg/mL, respectively.

Morphological changes imply that the combination of polymyxin B and nitazoxanide might cause dysfunction in cytoplasmic membrane. To test this, a DiSC3(5) probe was used to evaluate the inner membrane depolarization of *E. coli* cell (29). As shown in Fig. 5A, polymyxin B treatment resulted in a 1.5-fold enhancement of cell fluorescence compared to solvent treatment in DiSC3(5)-probe-labeled cells. Conversely, nitazoxanide treatment resulted in a nearly twofold lower fluorescence intensity of DiSC3(5)-probe-labeled cells compared to solvent treatment. Notably, the combined treatment with polymyxin B and nitazoxanide strongly reduced the fluorescence intensity of the labeled cells, similar to the treatment with nitazoxanide alone. The contrast in fluorescence intensity implies a different mechanism for the effect of polymyxin B and nitazoxanide on the electric potential (Δψ) of *E. coli*, with the effect of nitazoxanide prevailing over that of polymyxin B.

Previous studies demonstrated that membrane depolarization is directly involved in energizing key cellular processes such as calcium influx and ATP synthesis (30, 31). The concentration of intracellular calcium ions was detected with the Fluo-4 AM probe. As shown in Fig. 5B and C, *E. coli* cells treated with polymyxin B and treated with solvent both emitted green fluorescence, which was produced by the binding of influx calcium ions to the Fluo-4 AM probe. The calcium ion influx was due to cellular depolarization. Compared to the significant fluorescence intensity in bacteria treated with solvent, polymyxin B led to increased fluorescence, implying that membrane disruption and depolarization were exacerbated. However, the green fluorescence emitted by the binding of calcium ions to the probe was barely visible in cells treated with nitazoxanide alone or with a combination of polymyxin B and nitazoxanide. The blocking effect of nitazoxanide on calcium ion influx was consistent with the DiSC3(5)-probe assay. Furthermore, the ATP levels of *E. coli* cells exposed to polymyxin B in the presence and absence of nitazoxanide were studied. As shown in Fig. 5D, the treatment of polymyxin B alone resulted in a significant increase in cellular ATP levels by nearly twofold compared to the blank control group. In contrast, nitazoxanide resulted in a significant decrease in ATP levels. Similarly, the combination of polymyxin B and nitazoxanide resulted in a dramatic decrease in ATP levels. The changes in ATP with the substances were in agreement with calcium ion influx and the DiSC3(5)-probe assay.

ROS have recently been recognized as a common bactericidal mechanism for antibiotic activity. As shown in Fig. 5E, the fluorescence intensity responded to the amount of ROS with a mild increase in *E. coli* cells treated with polymyxin B alone. However, visible increases in ROS levels were observed in the cells treated with nitazoxanide alone. Similarly, polymyxin B combined with nitazoxanide significantly stimulated ROS accumulation in *E. coli* cells.

### Efficacy of nitazoxanide in mice models

**Fig 6:**
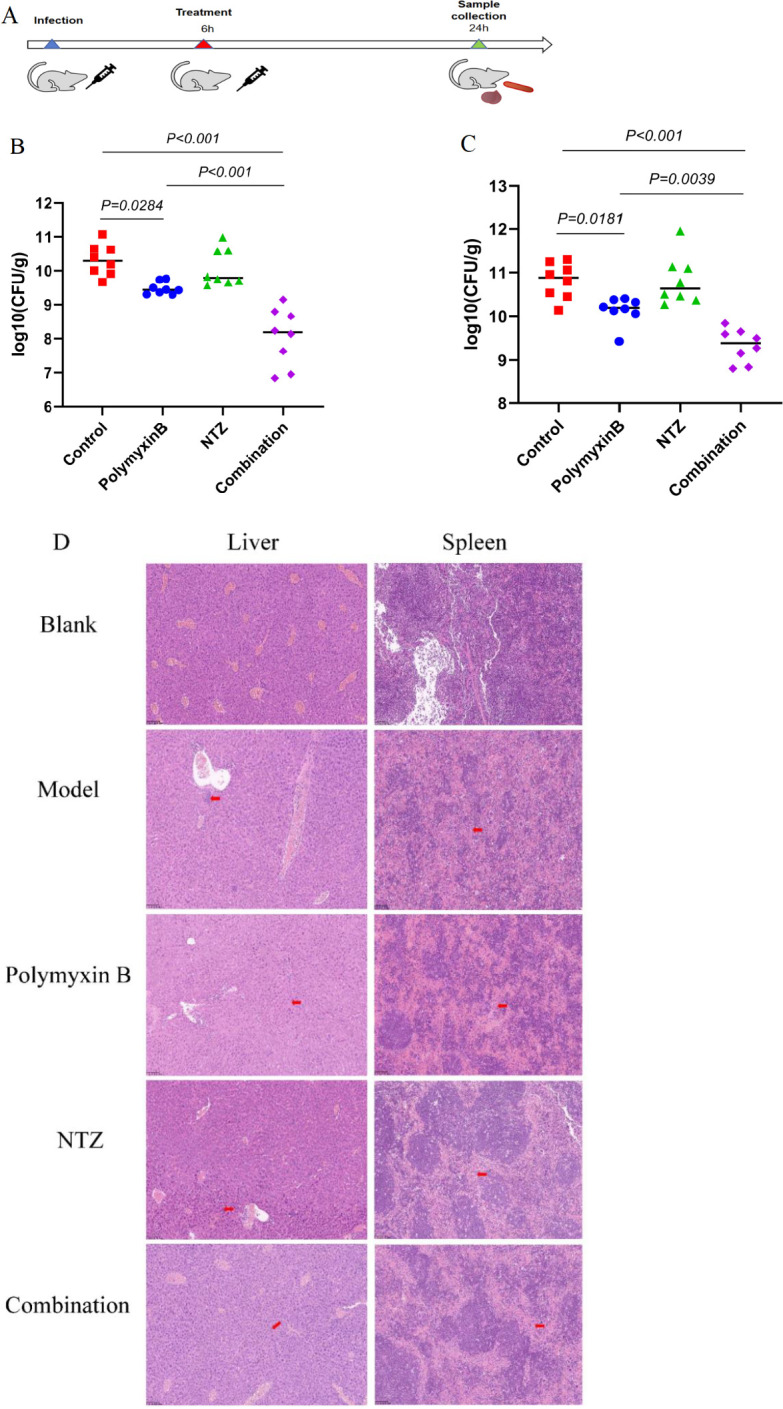
Therapeutic effects of mouse peritonitis/sepsis with the combination of polymyxin B and NTZ. The combination of NTZ and polymyxin B significantly reduced bacterial load in the liver and spleen, and reduced the lesions caused by bacterial infection compared with polymyxin B alone. (**A**) Protocol of the peritonitis-sepsis model infected with *E. coli* ATCC 25922. Bacterial burden in the liver (**B**) and spleen (**C**) of infected mice. (**D**) Histopathological morphology of murine tissues. The liver and spleen are in order from up to down.

The combination of polymyxin B and nitazoxanide exhibited a favorable synergistic antimicrobial effect *in vitro*, prompting further investigation into their therapeutic application in an animal model. The results depicted in Fig. 6A through C demonstrate that treatment with polymyxin B alone for a duration of 24 h significantly reduced *E. coli* colonization in both metabolic (liver) and immune (spleen) organs of mice. Treatment with nitazoxanide led to a decrease in bacterial colonization in the liver and spleen, although the reduction was not statistically significant. Encouragingly, the concurrent administration of polymyxin B and nitazoxanide demonstrated a noteworthy decrease of approximately two logarithmic units in colony-forming units (CFUs) when compared to the control group. Moreover, this combination treatment exhibited a greater reduction in bacterial colonization of the liver and spleen compared to the use of polymyxin B alone. The findings depicted in Fig. 6C further support these observations, as histopathological examination revealed a significant alleviation of hepatocellular edema and inflammatory cell infiltration, as well as a mitigation of splenic lymphocytopenia and red marrow congestion, both in the presence and absence of nitazoxanide during polymyxin B treatment. These *in vivo* effective results demonstrate the adjuvant potential of nitazoxanide to enhance polymyxin B in the treatment of bacterial infectious diseases.

### Transcriptomic analysis

**Fig 7:**
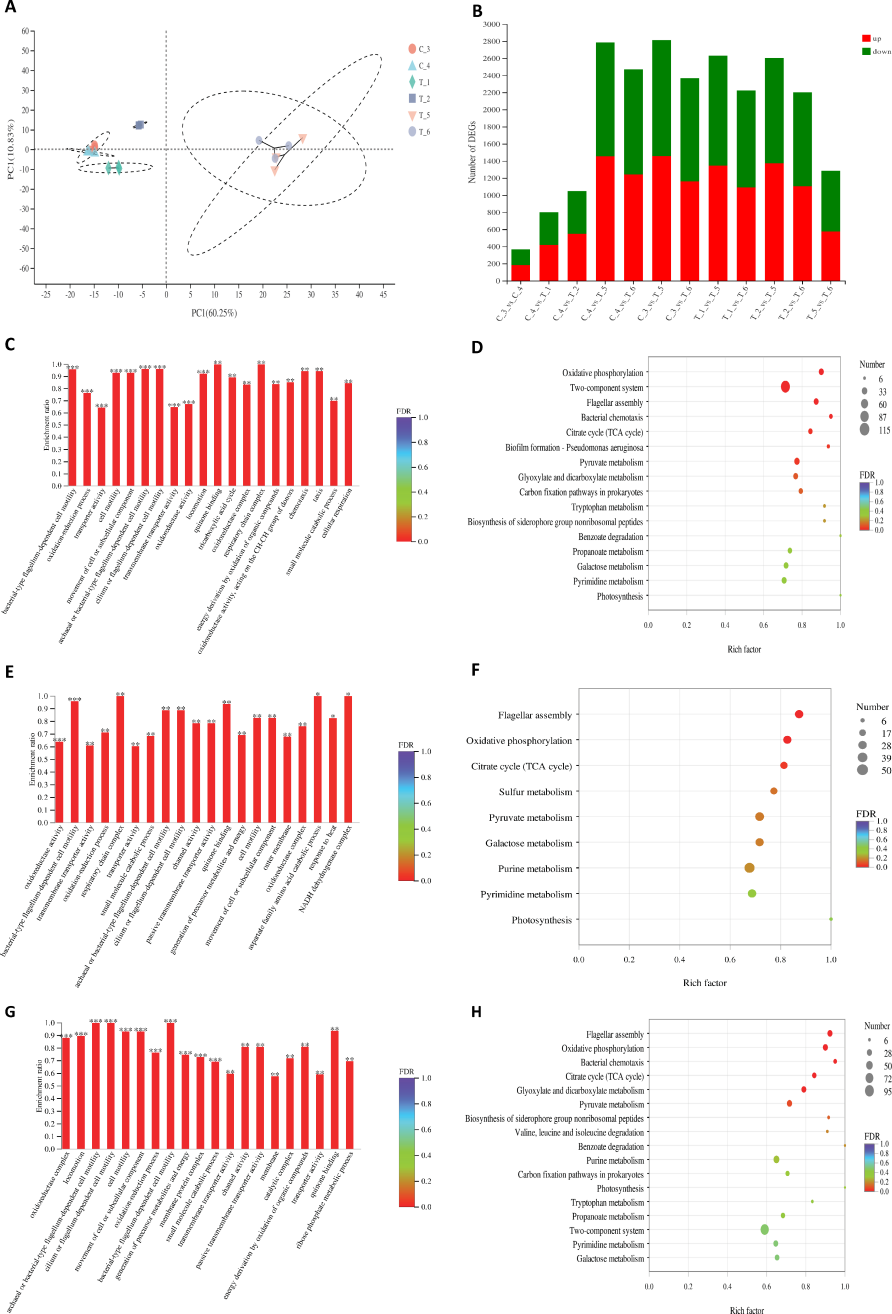
Transcriptome analysis of *E. coli* ATCC 25922 after exposure to polymyxin B, nitazoxanide, or the combination of polymyxin B–nitazoxanide. (**A**) PCA showed that the clustering of internal samples of transcriptional genes in each group is very tight, while the separation between the combined group (T_5 and T_6) and other groups (C_3, C_4, T_1 and T_2) is very obvious. (**B**) Bar graph showing a large number of DEGs. The X-axis represents the different treatment groups, and the number of DEGs changes on the Y-axis. Red indicates upregulation, while green indicates downregulation. (**C**) and (**D**) are GO and KEGG enrichment analysis of DEGs of C_4 compared with T_5; (**E**) and (**F**) are GO and KEGG enrichment analysis of DEGs of T_1 compared with T_5; (**G**) and (**H**) are GO and KEGG enrichment analysis of DEGs of T_2 compared with T_5, respectively. T_1 was treated with 0.5 µg/mL polymyxin B for 2 h, T_2 was treated with 16 µg/mL nitazoxanide for 2 h, T_5 was treated with a combination of 0.5 µg/mL polymyxin B and 16 µg/mL nitazoxanide for 2 h, T_6 was treated with a combination for 0.5 h, C_3 was the blank for 0 h, and C_4 was the blank for 2 h, respectively.

To gain a deeper understanding of the molecular mechanisms of nitazoxanide, we performed transcription analysis of *E. coli* after exposure to polymyxin B or the combination of polymyxin B and nitazoxanide. The principal component analysis (PCA) plot (Fig. 7A) indicated that there was a remarkable discrimination in detected transcriptional genes between blank groups (C_3 and C_4), polymyxin B (T_1) or nitazoxanide (T_2) groups, and combination groups (T_5 and T_6). The two blank groups (C_3 and C_4) are tightly clustered, while the two combination groups (T_5 and T_6) are loosely clustered, and the polymyxin B and nitazoxanide groups (T_1 and T_2) are completely separated. Further analysis revealed that a total of 2,636 differentially expressed genes (DEGs) were detected in pairwise comparisons (Fig. 7B), among which only 93 DEGs were screened in the C_4 group compared to the C_3 group, including 48 upregulated and 45 downregulated; compared to the combination treated for 2 h (T_5), the combination treated for 0.5 h (T_6) resulted in the upregulation of 110 DEGs and the downregulation of 310 DEGs. However, compared with the blank group (2 h, C_4), a total of 269, 260, 1,602, and 1,356 DEGs were screened in the polymyxin B (T_1), nitazoxanide (T_2), and two combination groups (2 h, T_5 group and 0.5 h, T_6 group), respectively. This result indicated that the different treatments caused a large change in the gene expression level of *E. coli*.

Focused on analyzing the DEGs in the blank treatment (2 h, C_4) vs combination treatment (2 h, T_5) group, the DEGs screened in the C_4 vs T_5 group included 858 upregulated genes and 744 downregulated genes (Fig. 7B). Gene Ontology (GO) enrichment analysis showed that these DEGs are remarkably correlated with 10 molecular functions (false discovery rate [FDR] < 0.001), including bacterial-type flagellum-dependent cell motility, oxidation-reduction process, transporter activity, cell motility, movement of cell or subcellular component, archaeal or bacterial-type flagellum-dependent cell motility, cilium or flagellum-dependent cell motility, transmembrane transporter activity, oxidoreductase activity, and locomotion (Fig. 7C). Kyoto Encyclopedia of Genes and Genomes (KEGG) enrichment analysis demonstrated that these DEGs were significantly enriched in oxidative phosphorylation with FDR < 0.001, and in two-component system, flagellar assembly, bacterial chemotaxis with FDR < 0.01 (Fig. 7D).

The comparison of treatment with the combination (T_5 group) to polymyxin B alone (T_1 group) revealed an upregulation of 723 and downregulation of 694 DEGs (Fig. 7B). GO enrichment analysis showed that these DEGs are remarkably correlated with oxidoreductase activity and bacterial-type flagellum-dependent cell motility (FDR < 0.001) (Fig. 7E). KEGG enrichment analysis demonstrated that these DEGs were significantly enriched in flagellar assembly (FDR < 0.001), oxidative phosphorylation (FDR < 0.01), and citrate cycle (TCA cycle) (FDR < 0.05) (Fig. 7F).

The comparison of treatment with the combination (T_5 group) to nitazoxanide alone (T_2 group) revealed an upregulation of 761 and downregulation of 676 DEGs (Fig. 7B). GO analysis showed that these DEGs are remarkably enriched in 11 molecular functions with FDR < 0.001, including oxidoreductase complex, locomotion, archaeal or bacterial-type flagellum-dependent cell motility, cilium or flagellum-dependent cell motility, cell motility, movement of cell or subcellular component, oxidation-reduction process, bacterial-type flagellum-dependent cell motility, generation of precursor metabolites and energy, membrane protein complex, and small molecule catabolic process (Fig. 7G). KEGG enrichment analysis demonstrated that these DEGs were significantly enriched in flagellar assembly, oxidative phosphorylation (FDR < 0.001), and bacterial chemotaxis, citrate cycle (TCA cycle), glyoxylate and dicarboxylate metabolism (FDR < 0.01) (Fig. 7H).

**Fig 8:**
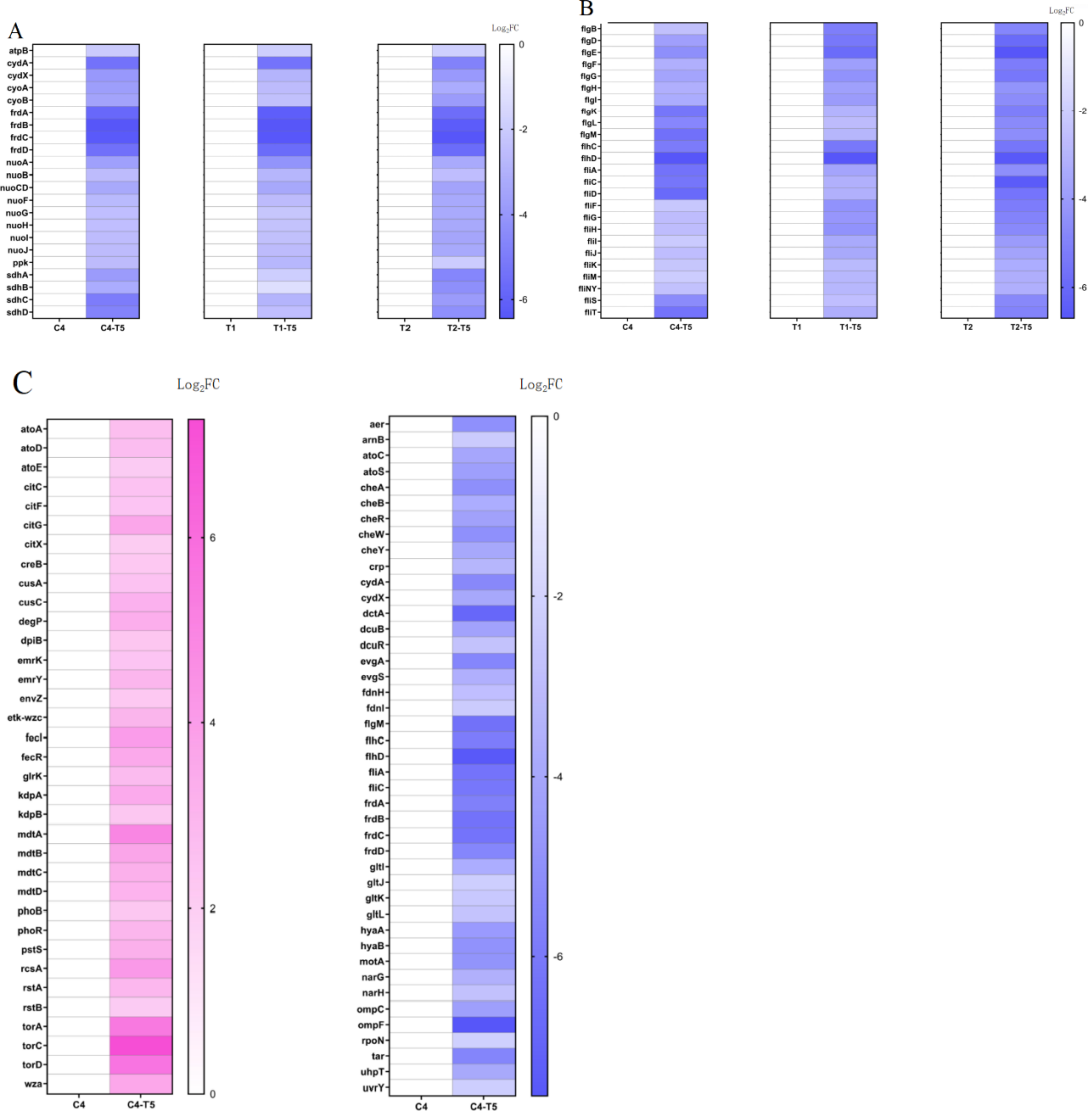
The expression of oxidative phosphorylation (**A**) and flagellar assembly functional genes (**B**) was significantly inhibited by the combined treatment. The two-component system (**C**) presented a bidirectional adjustment after the combined treatment. The combined action of polymyxin B and NTZ interfered the oxidative phosphorylation and ATP synthesis, and destructed the communication network in *E. coli* cells.

Obviously, the oxidative phosphorylation and flagellar assembly functional genes in bacteria were significantly downregulated under the combined treatment. Specifically, the gene expression of many oxidoreductase enzymes, including NADH-quinone oxidoreductase, fumarate reductase (*frd*), and succinate dehydrogenase (*sdh*), as well as the gene expression of the type III secretion system (T3SS), is significantly decreased (Fig. 8A). Notably, the genes related to the two-component system are both upregulated and downregulated (Fig. 8B), implying a disrupted function of quorum sensing in *E. coli* by combination treatment. Collectively, these data indicate that the combined action of polymyxin B and nitazoxanide leads to the interference of oxidative phosphorylation and ATP synthesis in *E. coli* cells and further leads to the destruction of the communication network, promoting bacterial cell death.

### The killing effect of polymyxin B on strain *E. coli* ATCC 25922 ∆*nuoC* was enhanced

**Fig 9:**
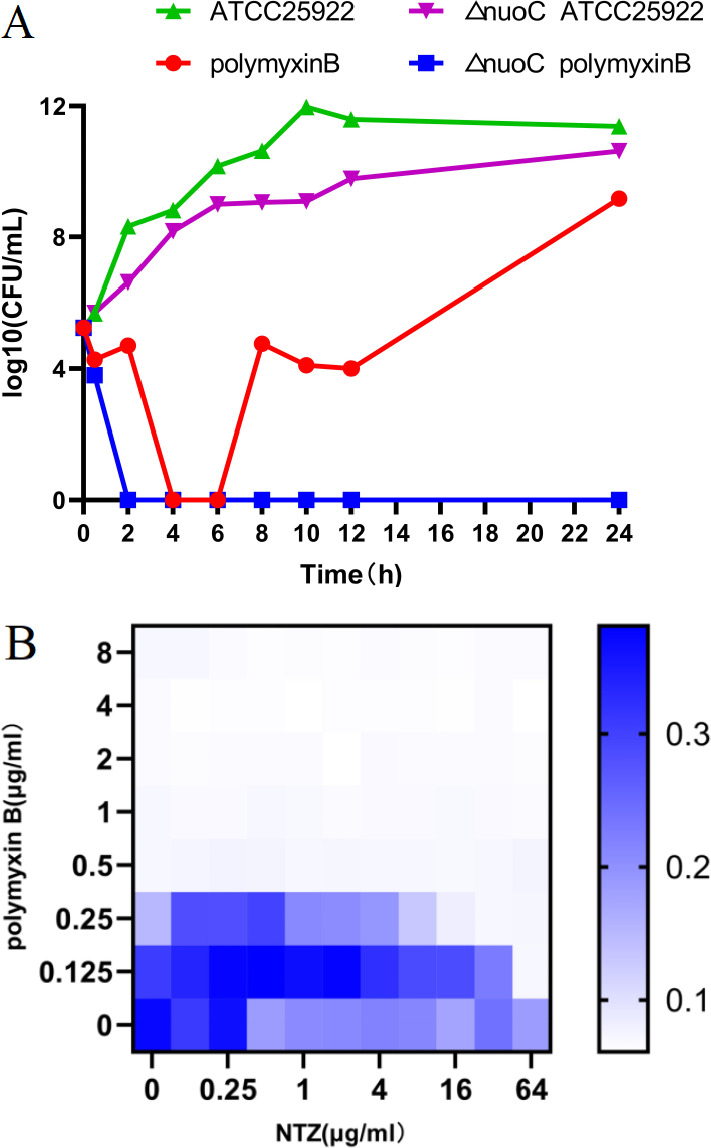
The strain *E. coli* ATCC 25922 ∆*nuoC* is more sensitive to polymyxin B than ATCC 25922. (**A**) The bactericidal curve; (**B**) checkerboard broth microdilution assays between NTZ and polymyxin B against the strain *E. coli* ATCC 25922 ∆*nuoC* (FIC = 1). Dark‐blue regions represent higher cell density and lower inhibition rate of combinational treatment. Data represent the mean OD (600 nm) of two biological replicates. *x*‐ and *y*‐axes of figures were presented as log2 scale. Synergy is defined as an FIC index of ≤0.5.

Joint analysis of affinity chromatography results (data not shown) and transcriptomic analysis results identified two potential target genes, namely *nuoC* and *fliC* (data not shown). In view of the close association of NuoC with oxidative phosphorylation, the red homologous recombination system was used to knock out the *nuoC* gene and construct the *E. coli* ATCC 25922 ∆*nuoC* (data not shown). In Mueller‐Hinton broth (MHB) medium, the growth of strain *E. coli* ATCC 25922 ∆*nuoC* was slightly slower than that of *E. coli* ATCC 25922 (Fig. 9A). The sub-MIC polymyxin B significantly inhibited the growth of *E. coli* ATCC 25922 within 6 h, and this inhibitory effect disappeared after 6 h. However, the strain *E. coli* ATCC 25922 ∆*nuoC* was completely killed after being treated with sub-MIC polymyxin B for 2 h. The bactericidal curve of ATCC25922 ∆*nuoC* treated with polymyxin B alone is very similar to that of ATCC25922 treated with a combination of polymyxin B and nitazoxanide. Furthermore, the FIC assay revealed that the synergistic effect of polymyxin B and nitazoxanide on strain *E. coli* ATCC 25922 ∆*nuoC* disappeared (FIC > 0.5, Fig. 9B), suggesting that the synergistic effect of nitazoxanide requires the involvement of NuoC or related proteins.

## DISCUSSION

Here, we demonstrate for the first time that the MIC value of the lipopeptide antibiotic polymyxin B against gram-negative bacteria can be significantly reduced by 4- to 16-fold in the presence of the anthelmintic drug nitazoxanide, indicating a selective synergistic effect (with FIC ≤ 0.5) between nitazoxanide and polymyxin B (Fig. 1; Table 1). Further tests revealed a synergistic effect of nitazoxanide in combination with polymyxin B against sensitive strains, resistant strains, and clinical isolates of *E. coli*, *S.* Typhi, *P. mirabilis*, and *K. pneumoniae* (Table 1). Additionally, we demonstrate that the elimination time of the clinical isolates of *E. coli* B2 by combination drugs was retarded by twice as much as standard sensitive strain in time-killing assays (Fig. 2A and B). The result indirectly confirmed that drug-resistant strains not only increased the required colistin concentration to inhibit growth but also reduced the rate of bacterial lysis (18). It should be noted that if the medium contained a certain amount of polymyxin B, *E. coli* passed continuously in the medium could acquire resistance to polymyxin B, regardless of whether the medium contained nitazoxanide. However, the development of resistance in the combination was later than that of polymyxin alone, and the combination still had a synergistic effect on the resulting polymyxin B-resistant strains (Fig. 2C). Due to widespread resistance among clinical isolates, the effectiveness of polymyxins is decreasing (11, 12). As a new adjuvant strategy, the synergistic effect of the combination of nitazoxanide and polymyxin B against polymyxin B-resistant bacteria undoubtedly provides the possibility to improve the effectiveness of polymyxin B and prolong the service life of polymyxin B. More importantly, using a mouse model of peritonitis/septicemia infected with *E. coli in vivo*, we found that the combination of nitazoxanide and polymyxin B reduced the bacterial load and lesions in the mouse organs more significantly than polymyxin B treatment alone (Fig. 6), suggesting that the combination has great clinical application prospects. Although *in vivo* polymyxin B treatment alone remains significantly effective, the potential for combination therapy provides a new means of countering polymyxin B resistance and defending polymyxin B as a “last resort” antibiotic for infections caused by gram-negative bacteria.

In the current study, nitazoxanide demonstrated to not only significantly enhance the inhibitory effect of polymyxin B on biofilm formation, but also significantly improve the permeability of polymyxin B to *E. coli* cell membrane (Fig. 3). The role of nitazoxanide in promoting polymyxin B to damage the integrity of *E. coli* cell membranes was further confirmed by visual comparison using TEM (Fig. 4). Polymyxin B causes the outer membrane to penetrate forward by replacing divalent cations responsible for stabilizing the integrity of the outer membrane, such as Ca^2+^ and Mg^2+^, leading to the collapse of the outer membrane and cell death before depolarization of the cell plasma membrane (10, 32, 33). Nevertheless, the inhibition of cell membrane depolarization and blockade of calcium ion influx exhibited by nitazoxanide in the present study appear to be in contradiction with the known mechanism of polymyxin B (Fig. 5). It is well known that polymyxin B is a strong cation lipopeptide that interacts closely with lipopolysaccharide molecules, particularly with lipid a and phosphate groups on the lipopolysaccharide core (10). Sufficient negatively charged components of the bacterial membrane are necessary for the interaction of polymyxin B with the membrane (34). The increase in positive charge on the surface of the bacterial outer membrane induced by modification of lipopolysaccharide is one of the colistin resistance mechanisms in gram-negative bacteria (35). Nitazoxanide displays considerable structural similarity with niclosamide and shares similar pharmacodynamic properties with niclosamide, such as disruption of bacterial membrane potential and intracellular pH homeostasis (36). It was previously reported that niclosamide increased the negative surface charge of *A. baumannii* and *K. pneumoniae* strains, and contributed to the restoration of colistin activity against gram-negative bacteria (37). Nuclear magnetic resonance studies identified two apparent forms of nitazoxanide: a biologically active anion (p*Ka* = 6.18) and a biologically inactive protonated form of NTZ at lower pH (38). Thus, the biologically active anions of nitazoxanide inhibit cell membrane depolarization and calcium ion influx, which may contribute to strengthen the surface anionicity of bacterial outer membranes. The increased anion enhances the affinity of polymyxin B to the outer membrane of gram-negative bacteria and further promotes higher outer membrane permeability.

It is well established that the antibacterial effect of polymyxin B on gram-negative bacteria is believed to involve multiple modes of action, such as destroying the bacterial outer membrane, causing the exchange of phospholipids between both membranes, and inhibiting vital respiratory enzymes (32, 39). As a potent inhibitor of the pyruvate ferredoxin oxidoreductase, nitazoxanide exhibits a broad-spectrum activity *in vitro* against strictly anaerobic bacteria (40), including *Clostridium difficile*, and against members of the Epsilonproteobacteria, including *Helicobacter pylori* and *Campylobacter jejuni* (38, 41). In contrast, previous studies have shown that Enterobacteriaceae are generally insensitive to nitazoxanide (28, 42), due to the lack of pyruvate ferredoxin oxidoreductase in bacteria such as Enterobacteriaceae, despite nitazoxanide inhibiting the biofilm formation mediated by aggregative adhesion fimbriae in enteroaggregative *E. coli* (43, 44). Our ultrastructural findings suggest that polymyxin B combined with nitazoxanide may have the potential to interact with cytoplasmic components (Fig. 4). Further research reveals that drug combinations significantly inhibit ATP synthesis and lead to ROS accumulation (Fig. 5). The consequence is mainly attributed to the contribution of nitazoxanide, due to the effect of nitazoxanide alone being significantly stronger than that of polymyxin B. The disruption of the proton motive force and the inhibition of ATP production by nitazoxanide in gram-negative bacteria caused an intracellular survival stress. Moreover, the intracellular ROS-induced oxidative stress produced by nitazoxanide caused further damage to the bacteria.

The effective production of ATP through oxidative phosphorylation is an absolute requirement for the survival of aerobic organisms (45). When aerobic respiration is blocked, bacteria may need to choose anaerobic respiration in order to maintain energy supply. Given the broad-spectrum activity of nitazoxanide against anaerobic bacteria, we hypothesized that polymyxin B disrupts gram-negative bacterial cell membranes leading to impaired aerobic respiration, and that the bacteria have to seek to utilize anaerobic respiratory modes for energy supply. However, the presence of nitazoxanide leads to anaerobic dysfunction, causing bacterial death due to energy depletion. By transcriptomic analysis, we found a significant decrease in the expression of genes related to oxidative phosphorylation function, which is consistent with the significant decrease in ATP production observed previously. Notably, the combination significantly downregulated the expression of NADH-quinone oxidoreductase (*nuoA-J*), cytochrome ubiquinol oxidase (*cydA*, *cydX*, *cyoA*, *cyoB*), fumarate reductase (*frdA-D*), and succinate dehydrogenase (*sdhA-D*) genes (Fig. 8). Fumarate reductase and succinate dehydrogenase represent a paired system involved in the anaerobic respiration of *E. coli* (46–48). Thus, the significant inhibition of fumarate reductase and succinate dehydrogenase gene expression indicates that the initiation of anaerobic respiration fails in *E. coli*, and that both aerobic and anaerobic modes of energy supply to the bacterium are blocked by the combination of polymyxin B and nitazoxanide. On the other hand, T3SS uses proton motive force and ATP hydrolysis to energize protein translocation (49). The suppression of almost all gene expression in T3SS corroborates that the drug combination resulted in energy depletion, proton motive force, and membrane disruption.

NuoC is a major member of NADH-quinone oxidoreductase and a major constituent subunit of oxidative phosphorylation complex I. A combined analysis of affinity chromatography results from our previous study (data not shown) and transcriptome analysis results from this study identified *nuoC* as a potential target gene. Constructing *nuoC* null strains by gene knockout will help to verify whether the action of nitazoxanide is accomplished through oxidative phosphorylation. The bactericidal effect of polymyxin B alone on strain *E. coli* ATCC 25922 ∆*nuoC* is consistent with that of polymyxin B and nitazoxanide combined treatment of *E. coli* ATCC 25922. This reveals that NuoC is a promising drug target for nitazoxanide, and also proves that the mechanism of nitazoxanide enhancing the bactericidal effect of polymyxin B is the inhibition of electron transfer and ATP production.

**Fig 10:**
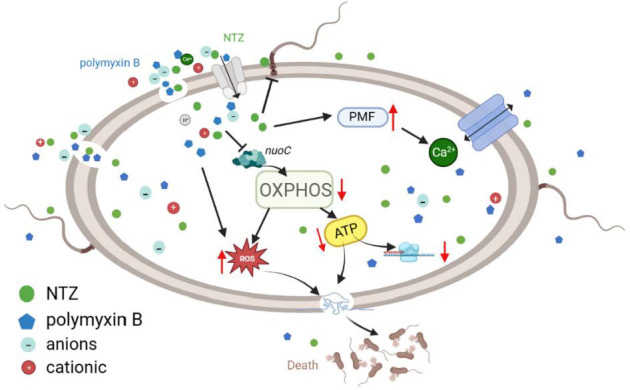
Scheme of synergistic mechanisms of NTZ in combination with polymyxin B against gram-negative bacteria. The biologically active anions of nitazoxanide enhance the membrane potential and surface anion strength of the plasma membrane, thereby promoting the membrane-breaking effect of polymyxin B. In addition, NTZ inhibits NuoC and oxidative phosphorylation, leading to ROS rise and ATP depletion. Multiple synergistic mechanisms enabled NTZ to enhance polymyxin B to kill gram-negative bacteria.

In conclusion, the anthelmintic drug nitazoxanide may serve as a potential adjuvant for polymyxin B therapy. There may be a new mode of action in the combination of nitazoxanide and polymyxin B, which disrupts bacterial oxidative phosphorylation function through dissipation of bacterial membrane potential, thereby depleting cellular energy and leading to cell death (Fig. 10). The combination of nitazoxanide with polymyxin B not only deepens our understanding of the versatile ability of nitazoxanide, but also provides an arsenal to treat infections of all MDR gram-negative bacteria.

## MATERIALS AND METHODS

### Bacteria and reagents

The bacterial strains used in this study are listed in Table 1. Strains were grown in MHB (Solarbio, China) or on MH agar (MHA) plates at 37°C. Unless otherwise noted, all experiments with *E. coli* were performed with ATCC 25922, or colistin-resistant strain B2 presented by Professor Kui Zhu (China Agricultural University, Beijing, China). Polymyxin B was obtained from Beijing Solarbio Science & Technology Co., Ltd. (Beijing, China). NTZ (≥98.5%) was synthesized by our laboratory and characterized by LC-UV, LC-MS, and NMR methods. All chemicals were of reagent grade quality or better, and were obtained from commercial suppliers.

### Antibacterial tests and checkerboard studies

*In vitro* antibacterial activity of the tested agents was assessed using the broth microdilution method according to the CLSI guidelines (50). The MIC for enrofloxacin against *E. coli* ATCC 25922 was determined for each test to serve as an internal quality control. Synergy *in vitro* was determined using checkerboard assays as described previously (13). Briefly, the antibiotic of interest was twofold serially diluted along the abscissa, whereas nitazoxanide was twofold serially diluted along the ordinate in a 96-well plate. Bacteria cultured overnight were diluted and added to each well with a final concentration of 5 × 10^5^ CFU/mL according to the bacterial dilution method described in CLSI guidelines. After being incubated at 37°C for 18 h, the 96-well plates were examined for visible turbidity. The FIC index was calculated using the formula: FIC index = (MIC of antibiotic in combination) / (MIC of antibiotic alone) + (MIC of nitazoxanide in combination) / (MIC of nitazoxanide alone). Synergy is defined as an FIC index ≤ 0.5. In this study, the FIC for non-antibacterial agent against *E. coli* in the combination was calculated to be zero (e.g., 1 ÷ > 256 = 0) when non-antibacterial agent did not show any antibacterial activity alone against bacteria at the highest concentration tested (e.g., 256 µg/mL), but antibacterial activity was observed when the compounds were tested in combination. DRI was used to evaluate the difference between the antibacterial doses in combination in comparison to their individual doses (14). DRI was calculated as follows: DRI = MIC of drug alone/MIC of drug in combination.

### Time-killing assays

The colonies of *E. coli* (ATCC 25922 and colistin-resistant strain B2) from overnight MHA plates were separately emulsified in normal sterile saline and were adjusted to an OD600 of 0.40 (equivalent to approximately 1~2 × 10^8^ CFU/mL). Subsequently, the bacterial suspensions were further supplemented with fresh MHB for a 100-fold dilution in order to obtain an initial concentration of 10^6^ CFU/mL. The selected combination and corresponding single-agent concentrations, as per the checkerboards, were set as combination therapy groups and monotherapy groups, whereas the control groups were treated without drugs but with 1.0% (vol/vol) dimethyl sulfoxide (DMSO). At each timepoint (0, 0.5, 2, 4, 8, 12, and 24 h), the cultures for each treatment group were collected, serially diluted 10-fold, and plated onto agar to count bacterial numbers after incubation at 37°C for 24 h.

### Emergence of resistance/serial passage assay

The emergence of resistance/serial passage assay was determined as previously described with minor modifications (51). *E. coli* ATCC 25922 cultured overnight were diluted into fresh MHB media supplemented with drugs, which were combination (0.5 µg/mL polymyxin B + 16 µg/mL NTZ) or corresponding single-agent concentrations. After being cultured at 37°C for 24 h, this culture was diluted into the adjusted corresponding concentration of drugs for the next passages. The process was repeated for 30 d, and the FIC index was calculated at 0, 1, 3, 6, 10, 15, 20, 25, and 30 passages.

### Biofilm inhibition assay

The crystal violet staining methods were used to assess the activities of polymyxin B in the presence and absence of nitazoxanide against biofilm inhibition in 96-well plates (52). Vancomycin was used as the positive control. *E. coli* ATCC 25922 cultured overnight were diluted into fresh MHB media supplemented with drugs at combination or corresponding single-agent concentrations. After being cultured at 37°C for 24 h, the medium was removed, and the well was gently washed three times with phosphate-buffered saline (PBS). Ethanol (100 µL) was added to each well for bacterial fixation for 20 min. The plates were incubated with 100 µL of 1% crystal violet for 5 min, after discarding the methanol and drying in air. Next, excess crystal violet was discarded, and then the plates were washed three times with PBS. Finally, 200 µL of 33% glacial acetic acid was added to the plates, followed by shaking for 15 min to dissolve the biofilm. The OD value of each well was measured at 570 nm using a microplate reader. Biofilm formation = (OD of drug/OD of blank control) × 100%.

### PI uptake assay

The suspension of 10^6^ CFU/mL *E. coli* was cultured in MHB media with drugs, which were combination or corresponding single-agent concentrations for 2 h. PBS was used for washing and resuspending *E. coli* cells. PI was then added at 4°C for 25 min, and DAPI under normal temperature for 15 min in the dark, respectively. The resuspending *E. coli* cells were visualized by Axio observer Z1 inverted fluorescence microscopy (Zeiss, Germany) and were analyzed by flow cytometry.

### Swimming motility assay

The swim motility assay was performed as previously described (53). Briefly, a molten medium was mixed with drugs and then poured into the six-well plates. The cultures of exponentially growing *E. coli* were harvested by centrifugation. After discarding the supernatants, the pellets were resuspended and adjusted to an OD600 of 0.40. After the agar media solidified, 10 μL of the suspension was point-inoculated into the center of each well and was incubated at 37°C. The ruler measured the bacterial swimming diameter at 24, 36, and 48 h. The experiment was repeated three times independently.

### Transmission electron microscopy characterization

After treatment with polymyxin B, nitazoxanide alone, or the combination for 2 h, *E. coli* was collected and fixed in 2.5% glutaraldehyde solution overnight at 4°C, followed by washing thrice with PBS. The samples were dehydrated by a graded series of concentrations of ethanol (50%, 70%, and 90%) for 10 min and then transferred to a mixture (vol/vol = 1:1) of 100% ethanol and acetone for 10 min. Subsequently, the acetone in the sample was continuously displaced by a graded series of concentrations of absolute acetone and epoxy resin (acetone:epoxy resin ratio of 1:1, 1:2, 1:3, and total epoxy resin). Finally, the specimens were sectioned with an ultramicrotome, stained with uranyl acetate and lead citrate, and observed using TEM (Thermo Fisher, USA).

### Membrane depolarization assay

The inner membrane depolarization in the presence of drugs was determined using a membrane potential-dependent probe, DiSC3(5) (54). Briefly, bacterial cells were washed and resuspended to obtain an OD600 of 0.2 with 5 mM HEPES buffer containing 20 mM glucose. Next, the bacterial suspension and a final concentration of 1 µM DisC3(5) (in 5 mM HEPES with 20 mM glucose and 0.1 M KCl) mixtures were added to 96-well black microplates and were incubated for 15 min. The probe-labeled *E. coli* cells were incubated with varying concentrations of drugs, and the change in fluorescence intensity was monitored with an interval of 30 min for 12 h at excitation and emission wavelengths of 622 and 670 nm, respectively.

### Intracellular calcium ions

After *E. coli* ATCC 25922 was incubated with the drug for 2 h, the bacterial cells were collected and washed three times with PBS. The resuspended *E. coli* cells were stained with DAPI for 15 min, then stained with 0.5 µM Fluo-4 AM after cleaning, and incubated at 37°C for 1 h. Washed thrice with PBS, the bacterial cells were suspended and transferred to a slide for visualization under a LSM880 confocal laser scanning microscope (Zeiss, Germany).

### ATP determination

Intracellular ATP levels of *E. coli* ATCC 25922 were determined using an enhanced ATP assay kit (Beyotime, Shanghai, China). Briefly, *E. coli* cells grown overnight were washed and resuspended to obtain an OD600 of 0.5 with PBS. Bacterial cells were treated with polymyxin B in the presence or absence of nitazoxanide at 4°C for 5 min. After centrifugation, the bacterial precipitates were lysed by lysozyme, centrifuged, and the supernatant was used for measuring intracellular ATP levels. The detection solution of kit was added to a 96-well plate and were incubated at room temperature for 5 min. Subsequently, the supernatants were transferred into the well and were mixed quickly, and the ATP level was measured by Synergy H1 microplate reader (BioTek, USA) in the luminescence mode.

### Total reactive oxygen species (ROS) measurement

The resuspending *E. coli* cells were loaded 10 µM DCFH-DA fluorescent probe and were incubated in the dark at 37°C for 30 min following the manufacturer’s instruction (Beyotime, Shanghai, China). After centrifugation, the resuspending *E. coli* cells were incubated with polymyxin B in the presence and absence of nitazoxanide at 37°C for 4 h. Bacterial suspension (200 µL) was transferred into a well of 96-well plate with a black bottom. The levels of ROS in *E. coli* ATCC 25922 were immediately measured by the fluorescence intensity using Synergy H1 microplate reader (BioTek, USA) with the excitation wavelength at 488 nm and emission wavelength at 525 nm. Rosup was used as a positive control of ROS production.

### Mouse infection studies

Mice were used for this study. ICR male mice were purchased from SpePharm (Beijing) Biotechnology Co., Ltd., with eight animals per group. This study was approved by the Committee of Experimental Animal Center of Shanghai Veterinary Research Institute, Chinese Academy of Agricultural Sciences (No. SV-20220304-01).

*E. coli* ATCC 25922 cultured overnight was washed and resuspended to obtain an OD600 of 0.8 with physiological saline solution. Mice were infected with 300 µL *E. coli* ATCC 25922 suspension via intraperitoneal injection. At 6 h post-infection, the mice were treated with a specified intraperitoneal administration of PBS, polymyxin B (1.5 mg/kg), nitazoxanide (24 mg/kg) alone, or a combination of polymyxin B with nitazoxanide (1.5 mg/kg  + 24 mg/kg). The state of the mice was observed and recorded during the experiment. After 24 h of treatment, the liver and spleen were collected to analyze the bacterial load (53, 55). At the same time, the liver and spleen sections were fixed in 10% formaldehyde, embedded in paraffin, and stained with hematoxylin.

### Transcriptomic analysis

*E. coli* ATCC 25922 was grown in MHB to the exponential phase. Then, cells were divided into six treatment groups: T_1 was treated with polymyxin B (0.5 µg/mL) for 2 h, T_2 was treated with nitazoxanide (16 µg/mL) for 2 h, T_5 was treated with a combination of polymyxin B and nitazoxanide (0.5 µg/mL + 16 µg/mL) for 2 h, T_6 was treated with a combination for 0.5 h, C_3 was the blank for 0 h, and C_4 was the blank for 2 h, respectively. After incubation, cells were harvested, and the total RNA of samples was extracted using the TruSeq Stranded Total Library Preparation kit (Illumina, USA). RNA purity was determined by the ratio of OD260/280 using a Nanodrop spectrophotometer (Thermo Scientific), and RNA integrity was verified using 1.5% agarose gels. The samples were sequenced using the Illumina HiSeq 4000 system at Shanghai Majorbio Bio-pharm Biotechnology Co., Ltd. (Shanghai, China) with 200 cycles (2  ×  150 bp read length). Raw sequencing reads were subjected to filtration by quality control, and further analyses were performed on the free online platform of Majorbio Cloud Platform (Majorbio, Shanghai, China). Transcripts per million (TPM) were determined to quantify gene expression levels. After analyzing the gene expression levels, we established the screening criteria for DEGs based on the absolute value of fold change being ≥2 with FDR ≤ 0.001. We were able to visualize the significant DEGs using volcano curves and heatmaps. Additionally, we performed KEGG pathway and GO term enrichment analysis for the DEGs and made a comparative analysis between the treatment group and the control group.

### Sensitivity of *E. coli* ATCC 25922 ∆*nuoC* to polymyxin B

Strain *E. coli* ATCC 25922 ∆*nuoC* was obtained from our laboratory, which used the Red method to knock out the *nuoC* gene. Then, the time-killing assays and the FIC assays of *E. coli* ATCC 25922 ∆*nuoC* were performed using the method described earlier.

### Statistical analyses

Statistical analysis was performed using GraphPad Prism 8 software. All data are presented as mean  ±  SD.
